# Public Health and Air Pollution in Asia (PAPA): A Multicity Study of Short-Term Effects of Air Pollution on Mortality

**DOI:** 10.1289/ehp.11257

**Published:** 2008-07-09

**Authors:** Chit-Ming Wong, Nuntavarn Vichit-Vadakan, Haidong Kan, Zhengmin Qian

**Affiliations:** 1 Department of Community Medicine, School of Public Health, The University of Hong Kong, Hong Kong Special Administrative Region, China; 2 Faculty of Public Health, Thammasat University, Pathumthani, Thailand; 3 School of Public Health, Fudan University, Shanghai, China; 4 Epidemiology Branch, National Institute of Environmental Health Sciences, Research Triangle Park, North Carolina, USA; 5 Pennsylvania State University College of Medicine, Hershey, Pennsylvania, USA; 6 Geisinger Center for Health Research, Danville, Pennsylvania, USA

**Keywords:** air pollution, Bangkok, Hong Kong, mortality, Shanghai, time-series analysis, Wuhan

## Abstract

**Background and objectives:**

Although the deleterious effects of air pollution from fossil fuel combustion have been demonstrated in many Western nations, fewer studies have been conducted in Asia. The Public Health and Air Pollution in Asia (PAPA) project assessed the effects of short-term exposure to air pollution on daily mortality in Bangkok, Thailand, and in three cities in China: Hong Kong, Shanghai, and Wuhan.

**Methods:**

Poisson regression models incorporating natural spline smoothing functions were used to adjust for seasonality and other time-varying covariates that might confound the association between air pollution and mortality. Effect estimates were determined for each city and then for the cities combined using a random effects method.

**Results:**

In individual cities, associations were detected between most of the pollutants [nitrogen dioxide, sulfur dioxide, particulate matter ≤ 10 μm in aerodynamic diameter (PM_10_), and ozone] and most health outcomes under study (i.e., all natural-cause, cardiovascular, and respiratory mortality). The city-combined effects of the four pollutants tended to be equal or greater than those identified in studies conducted in Western industrial nations. In addition, residents of Asian cities are likely to have higher exposures to air pollution than those in Western industrial nations because they spend more time outdoors and less time in air conditioning.

**Conclusions:**

Although the social and environmental conditions may be quite different, it is reasonable to apply estimates derived from previous health effect of air pollution studies in the West to Asia.

Time-series studies of daily mortality in several Asian cities can contribute significantly to the world’s literature on the health effects of air pollution. First, they provide direct evidence of air pollution effects in areas for which there are few studies. Second, because they involve different exposure conditions and populations, mortality studies of Asian cities can shed light on factors that may modify the effects of air pollution on health. In addition, multicity collaborative studies conducted within Asia, especially when analyzed using a common protocol, can generate more robust air pollution effect estimates for the region than those from individual studies and provide relevant and supportable estimates of the local impacts of environmental conditions for decision makers. Finally, they can determine the appropriateness of applying the results of health effects of air pollution studies conducted in North America and Western Europe to regions where few studies, if any, have been conducted.

Recent reviews (Anderson et al. 2004; Ostro 2004) suggest that proportional increases in daily mortality per 10-μg/m^3^ increase in PM_10_ (particulate matter ≤ 10 μm in aerodynamic diameter) are generally similar among North American and Western European regions and the few developing countries where studies have been undertaken. However, the relatively few studies that have been conducted in Asia are not geographically representative and have used different methodologies, making it difficult to compare results in Asian cities with each other or with the broader literature. In addition, the worldwide data have not been appropriately analyzed for real differences in the magnitude of the effects of short-term exposure and the possible reasons for such differences, such as sources of air pollution or population characteristics.

Efforts to bring the world’s data together for such analyses are under way with funding from the Health Effects Institute (HEI) in the PAPA (Public Health and Air Pollution in Asia) project and the APHENA (Air Pollution and Health: A European and North American Approach) project. These efforts can provide important insights to the time-series literature in terms of variability in air pollution, climate, population, and city characteristics involved.

The first phase of the PAPA study was carried out using data from Bangkok, Thailand, from 1999 to 2003, Hong Kong, China, from 1996 to 2002, and Shanghai and Wuhan, China, both from 2001 to 2004 (Figure 1) (HEI 2008). A common protocol (available from the authors) for the design and analysis of data from multiple Asian cities and a management framework to conduct the coordinated analysis were established. These were designed to provide a basis for combining estimates and for isolating important independent factors that might explain effect modification in the city-specific estimates. It is anticipated that the results will not only contribute to the international scientific discussion on the conduct and interpretation of time-series studies of the health effects of air pollution but will also stimulate the development of routine systems for recording daily deaths and hospital admissions for time-series analysis.

## Materials and Methods

### Mortality data

We focused on mortality from all natural causes in all ages, ≥ 65 years, and ≥ 75 years, and for cardiovascular and respiratory disease at all ages. The *International Classification of Disease*, *Ninth Revision* [ICD-9; World Health Organization (WHO) 1977] and *Tenth Revision* (ICD-10; WHO 1992) rubrics of the health outcomes were as follows: all natural causes, ICD-9 codes 001–799 or ICD-10 codes A00–R99; cardiovascular, ICD-9 codes 390–459 or ICD-10 codes I00–I99; and respiratory, ICD-9 codes 460–519 or ICD-10 codes J00–J98.

The sources of health data were the Ministry of Public Health, Bangkok; the Census and Statistics Department, Hong Kong; the Shanghai Municipal Center of Disease Control and Prevention, Shanghai; and the Wuhan Centre for Disease Prevention and Control, Wuhan.

### Air pollutant and meteorological data

Air quality indicators included nitrogen dioxide, sulfur dioxide, PM_10_, and ozone. For NO_2_, SO_2_, and PM_10_, daily data were 24-hr averages and an 8-hr average was used for O_3_ (1000–1800 hours). Each city maintains several fixed-site air monitoring stations—dispersed throughout the metropolitan areas—that met the quality assurance and quality control procedures of local governments. The air pollutant concentrations were measured in Bangkok by the Pollution Control Department, Ministry of Natural Resources and Environment (*n* = 10 air monitoring stations); in Hong Kong by the Environmental Protection Department (*n* = 8); in Shanghai by the Shanghai Environmental Monitoring Center (*n* = 6); and in Wuhan by the Wuhan Environmental Monitoring Center (*n* = 6). The measurement methods for NO_2_, SO_2_, and O_3_ were similar for the four cities based on chemiluminescence, fluorescence, and ultraviolet absorption, respectively, whereas for PM_10_, the Chinese cities used tapered element oscillating microbalance and Bangkok used beta gauge monitors.

The calculation of 24-hr average concentrations of NO_2_, SO_2_, and PM_10_, and 8-hr average concentrations of O_3_ required at least 75% of the 1-hr values on that particular day. If > 25% of the daily values were missing for the whole period of analysis, the entire station was not included for that particular pollutant. Missing data were not imputed.

### Statistical analysis

The analytical methods were developed and adopted by all four teams in a common protocol. The protocol includes the specifications for selection of monitoring stations, as well as quality assurance and quality control procedures for data collection and for health outcomes and air pollutants to be included in the analysis. Generalized linear modeling was used to model daily health outcomes, with natural spline smoothers (Burnett et al. 2004; Wood 2006) for filtering out seasonal patterns and long-term trends in daily mortality, as well as temperature and relative humidity. We also included an adjustment for the day of the week and dichotomous variables relevant to individual cities if available, such as public holidays (Hong Kong) and extreme weather conditions (Wuhan). In an attempt to minimize autocorrelation, which would bias the standard errors, the aim of the core model was for partial autocorrelation function plots to have coefficients in absolute values < 0.1 for the first 2 lag days. Randomness of residuals was also considered in selecting the most appropriate models. If these criteria were not met, other methods were used to reduce autocorrelation, such as the inclusion of explanatory variables to model influenza epidemics and the addition of autoregression terms. If there were special periods with extra variations for which the core model could not account, an additional spline smoother was included. Air pollutant concentrations were entered into the core model to assess the health effects of specific pollutants. Exposure at the current day (lag 0), a 2-day average of lag 0 and lag 1 days (lag 0–1), and a 5-day average of lag 0 to lag 4 days (lag 0–4) were examined. For each pollutant, the excess risk of mortality with the 95% confidence interval (CI) per 10-μg/m^3^ increase in average concentration at lag 0–1 was calculated. However, for brevity’s sake, point estimates with *p*-values could be used to describe sets of effects.

Because several differences were observed in effect estimates among cities, we conducted additional sensitivity analyses to attempt to explain these differences and to determine the robustness of the initial findings. We focused on PM_10_, given the wealth of worldwide findings of effects from this pollutant, and used the average concentration of lag 0–1 days. In these analyses we aimed to explore the impact of the following: higher concentrations of PM_10_ that might be dominated by the coarse fraction and therefore have differential toxicity; monitors that might be overly affected by proximity to traffic; effects of different seasonality patterns among the cities; different controls for temperature; and different ways in aggregating daily concentration data and differences in spline models. We regarded a change of excess risk > 20% from that of the analysis as an indication of sensitive results. Specifically, the sensitivity analysis included the following items:

- Exclude the daily concentration of PM_10_ > 95th percentile
- Exclude the daily concentration of PM_10_ > 75th percentile
- Exclude the daily concentration of PM_10_ > 180 μg/m^3^
- Exclude monitoring stations with high traffic sources (highest nitric oxide/nitrogen oxides ratio)
- Assess warm season effect with dummy variables of seasons in the core model
- Add temperature at average lag 1–2 days or 3–7 days into the model
- Use a centered daily concentration of PM_10_ (Wong et al. 2001)
- Use natural spline with degrees of freedom (df) of time trend per year, temperature, and humidity fixed at 8, 4, and 4, respectively
- Use penalized spline instead of natural spline.

Combined estimates of excess risk of mortality and their standard errors were calculated using a random-effects model. Estimates were weighted by the inverse of the sum of within-and between-study variance.

Concentration–response curves for the effect of each pollutant on each mortality outcome in the four cities were plotted. We applied a natural spline smoother with 3 df on the pollutant term. We assessed nonlinearity by testing the change of deviance between a nonlinear pollutant (smoothed) model with 3 df and linear pollutant (unsmoothed) model with 1 df.

The main analyses and the combined analysis were performed using R, version 2.5.1 (R Development Core Team 2007). We also used mgcv, a package in R.

## Results

Table 1 summarizes the mortality data for the four cities, and Table 2 summarizes the pollution and meteorological variables. The daily mortality counts for all natural causes at all ages for each city showed more marked seasonal variations in the cities farther north. Shanghai (mean daily deaths, 119; population, 7.0 million) and Bangkok (95; 6.8 million) had higher daily numbers of deaths than Hong Kong (84; 6.7 million) and Wuhan (61; 4.2 million). The ratios for causes of death due to cardiovascular disease relative to respiratory disease were the highest in Wuhan (4:1) followed by Shanghai (3:1), Bangkok (2:1), and Hong Kong (1.5:1). The proportion of total cardiorespiratory mortality was also the highest in Wuhan (57%) followed by Shanghai (49%), Hong Kong (48%), and Bangkok (23%) [Table 1; Supplemental Material, Table 1 (available online at http://www.ehponline.org/members/2008/11257/suppl.pdf)]. Deaths occurring at ≥ 65 years of age were less frequent in Bangkok (36%) than in the three Chinese cities (72–84%).

As indicated in Table 2 and Figure 2, Wuhan showed the highest concentrations of PM_10_ and O_3_, whereas Shanghai had the highest concentrations of NO_2_ and SO_2_. The latter was probably due to the significant local contribution of power plants in Shanghai’s metropolitan area. To provide an indication of the relative magnitude of the pollution concentrations in these four large Asian cities, we compared them to the 20 largest cities in the United States using data from 1987 to 1994 from the National Morbidity, Mortality, and Air Pollution Study (NMMAPS) (Samet et al. 2000). Generally, in the PAPA cities, the concentrations of PM_10_ and SO_2_ were much higher than those reported in the United States (PM_10_ means of 52–142 μg/m3 in the cities of the PAPA study vs. 33 μg/m^3^ in NMMAPS, and SO_2_ means of 13–45 μg/m3 vs. 14 μg/m^3^); comparisons of NO_2_ and O_3_ showed a fairly similar pattern.

**Figure 1:**
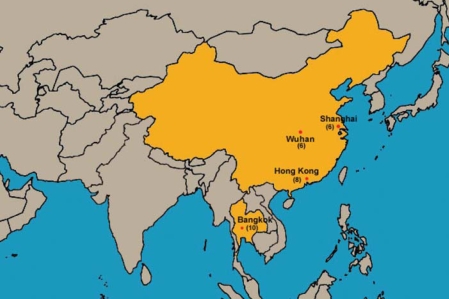
Bangkok, Hong Kong, Shanghai, and Wuhan. Numbers in parentheses indicate the number of monitoring stations used in each city.

We demonstrated the adequacy of the core models with partial autocorrelation function plots of the residuals in the previous 2 days, all within |0.1| [Supplemental Material, Figure 1 (available online at http://www.ehponline.org/members/2008/11257/suppl.pdf)].

In individual cities, for all natural causes at all ages (Table 3) the percentage of excess risk per 10-μg/m^3^ associated with NO_2_ ranged from 0.90 to 1.97 (all *p*-values ≤ 0.001); with SO_2_, from 0.87 to 1.61 (all *p*-values ≤ 0.05); with PM_10_, from 0.26 to 1.25 (all *p*-values ≤ 0.001); and with O_3_, from 0.31 to 0.63 (all *p*-values ≤ 0.05), but the effect in Wuhan was not significant. The excess risk showed trends of increasing risk with increasing age for all four pollutants. The trends for the age-specific effects were the strongest in Bangkok, less strong in Hong Kong and Wuhan, but absent in Shanghai (Figure 3). For all four pollutants, the excess risk in Bangkok was higher than those in the three Chinese cities. When the pollutant concentrations were expressed as the interquartile range (IQR; i.e., 75th percentile–25th percentile), Bangkok estimates were comparable to those of the three Chinese cities, particularly in all ages. Within cities, the effect estimates of different pollutants were also comparable to each other (data not shown).

In all cities, there was heterogeneity in effect estimates for NO_2_ and PM_10_ on all natural-cause mortality and for PM_10_ on cardiovascular mortality (Table 3). For all natural-cause mortality, the combined random effects excess risk were 1.23, 1.00, 0.55, and 0.38% for NO_2_, SO_2_, PM_10_, and O_3_, respectively (all *p-*values ≤ 0.05). The results for cardiovascular mortality (Table 3) followed a generally similar pattern, with the highest excess risk per 10-μg/m^3^ in Bangkok for PM_10_ and O_3_, and in Wuhan for NO_2_ and SO_2_. All of the cities demonstrated significant associations for each pollutant except SO_2_ in Bangkok and O_3_ in Wuhan, whereas all of the combined estimates were statistically significant. A similar pattern was shown for respiratory mortality, for which the highest estimates were found in Wuhan for NO_2_ and SO_2_ and in Bangkok for PM_10_ and O_3_. All the random effects estimates were statistically significant at the 5% level except for O_3_.

For the lag effects in the three Chinese cities, with a few exceptions, the average lag 0–1 days usually generated the highest excess risk. However, for Bangkok the longer cumulative average of lag 0–4 days generated the highest excess risk for all of the pollutants except SO_2_. For the combined estimates, effects at the lag 0–1 days showed the highest excess risk, except O_3_, for which the effect at lag 0–4 days was the greatest (data not shown).

Sensitivity analyses for PM_10_ showed that, in general, the results were fairly robust for various concentrations, monitors, specifications for temperature, methods of aggregating daily data, df used in the smoothers, and alternative spline models. In all cases, the effect estimates were statistically significant. In all cities, the effect estimates for PM_10_ were sensitive to exclusion of the higher concentrations. For the Chinese cities, this increased the excess risk > 20% for PM_10_, but in Bangkok the effect estimate decreased, with the excess risk changing from 1.25% to 0.73% per 10-μg/m^3^ increase in average concentration of lag 0–1 days (Table 4). Examination of the warm season (which varied for each city) resulted in significant increases in effect estimates for Bangkok and Wuhan but decreases in Hong Kong and, to a lesser extent, in Shanghai (excess risk changed from 0.26% to 0.24%). Adjusting for temperature through use of longer-term cumulative averages tended to decrease the PM_10_ effect.

The smoothed concentration-response (CR) relationship, between all natural-cause mortality and concentration of each pollutant, appeared to be positive. Most CR curves showed linear relationships over the IQR of the concentrations (Figure 4). At all ages, tests for nonlinearity for the entire curve showed that linearity could not be rejected at the 5% level for most of the associations between air pollution and mortality (data not shown).

## Discussion

### Review of PAPA project results

In the city-specific main effects for the five main health outcomes under study, there were variations in effect estimates between cities. For NO_2_ the estimates were similar in magnitude and precision for Bangkok and Wuhan, and for Hong Kong and Shanghai. The effects for Bangkok and Wuhan were higher but less precise (as reflected by a wider 95% CI) than for Shanghai and Hong Kong. For SO_2_ the estimates for Bangkok were higher but less precise than for the three Chinese cities. For PM_10_ the estimates in the three Chinese cities were very similar, but estimates were higher and less precise in Bangkok. For O_3_ the effect estimates and the precision among the four cities were similar, although estimates in Bangkok were higher. However, when expressed by IQR increase in concentrations, the effect estimates for each pollutant were similar in the four cities.

In the combined four-city analysis, the excess risks per 10-μg/m^3^ increase in NO_2_ were 2–3 times greater than those derived from the APHEA (Air Pollution and Health: A European Approach) project (Samoli et al. 2006) for mortality at all ages due to all natural causes, cardiovascular disease, and respiratory disease (1.23% vs. 0.3%, 1.36% vs. 0.4%, and 1.48% vs. 0.38%, respectively). For SO_2_, the estimate (random effects) of 1.00% for mortality due to all natural causes derived from the present study was higher than the 0.52% previously reported from the other Asian cities studied (HEI 2004) and higher than the 0.40% from the APHEA project (Katsouyani et al. 1997) [Supplemental Material, Table 2 (available online at http://www.ehponline.org/members/2008/11257/suppl.pdf)]. For PM_10_, the excess risk of 0.55% for all natural causes of death at all ages was comparable to 0.49% from all Asian cities (HEI 2004), 0.5% from NMMAPS (Samet et al. 2000), and 0.6% from the APHEA project (Anderson et al. 2004). A meta-analysis of Chinese studies found that each 10-μg/m^3^ increase in PM_10_ concentration was significantly associated with 0.3% increase in all natural-cause mortality, 0.4% increase in cardiovascular mortality, and 0.6% increase in respiratory mortality (Aunan and Pan 2004). For O_3_, the estimate from the present study was significant and higher than that from APHEA (Anderson et al. 2004) and NMMAPS (Bell et al. 2004) for all natural causes (0.38 vs. 0.20 and 0.26, respectively) and similar for cardiovascular causes (0.37 vs. 0.4 and 0.32); however, the estimates for respiratory disease (0.34 vs. −0.1 and 0.32%) were similar to those of the NMMAPS, but negative and statistically not significant (*p* > 0.05) in APHEA [Supplemental Material, Table 2).

### Review of estimates from previous Asian studies

For NO_2_, we found few time-series studies, and these were mainly from South Korea (Hong et al. 1999) and Hong Kong (Wong et al. 2001). The variation of effects was large compared with other pollutants for all natural-cause mortality, respiratory mortality, and cardiovascular mortality. For SO_2_, most time-series studies in China showed significant association with all natural-cause mortality, even at levels below the current WHO Air Quality Guideline (Chen et al. 2004; WHO 2005). A review of Asian studies (HEI 2004) also found that SO_2_ was associated with all natural-cause mortality either from random-effects models or fixed-effects models. For PM_10_, although fewer time-series studies were published from Asia than from other regions, most studies found a significant association with all natural-cause mortality, but only respiratory and cardiovascular mortality were examined in Bangkok (Ostro et al. 1999). However, significant associations with respiratory and cardiovascular mortality were not found in Seoul, Korea (Hong et al. 1999), or Hong Kong studies (Wong et al. 2001). For O_3_ studies using different time-average concentrations such as 1, 8, and 24 hr, the estimates varied greatly between studies (HEI 2004).

In the four individual cities included in the PAPA project, consistent with other studies for Asia, air pollution effects were found in each city and for all the disease-specific outcomes under consideration. The results provide important information on air pollution–related health effects in Asia, especially for areas known to have high exposures but are under-represented in the literature.

### Robustness of the results

Our sensitivity analyses indicated that most of the PM_10_ effect estimates did not deviate from the main analysis > 20%. The PM_10_ effect estimates were insensitive to different methods adopted, the use of higher df, and the replacement of the smoothing function by the penalized spline. However, across the four cities, additional adjustment for the average temperature at 3–7 lag days showed that the estimates for effects of PM_10_ were attenuated, indicating possible residual confounding due to uncontrolled lag effects of temperature. Studies (Schwartz et al. 2004; Medina-Ramón and Schwartz 2007) show that different cumulative lag days of temperature have effects on both morbidity and mortality estimates. However, in the present study, current day temperature was specified *a priori* in the core model and was determined to be sufficient to adjust for temperature effects at the beginning of the study. On the other hand, we found high correlations between temperatures at each lag 1–7 days and at the current day, which suggest problems of multicollinearity if we make further adjustment to these lag temperature effects in the model of the main analysis.

### Scientific issues derived from PAPA study results

For all natural-cause, cardiovascular, and respiratory mortality, the effect estimates of PM_10_ and O_3_ are relatively similar among the three Chinese cities. However, there are some differences in the PM_10_ effect estimates in that Shanghai is consistently lower, by almost half, than Hong Kong and Wuhan. These differences in effect estimates may be related to differences in the location of the monitoring stations and differences in the actual ambient levels of exposure of the population.

Estimates for PM_10_ in Bangkok were higher, and the effect estimates much higher, than those of the three Chinese cities (1.25 vs. 0.26–0.53; 1.90 vs. 0.27–0.61; and 1.01 vs. 0.27–0.87). The reasons might be related to consistently higher temperature, a population that spends a longer time outdoors, and less availablity and use of air conditioning in Bangkok than in the other cities (Ostro et al. 1999). With relatively higher mortality due to infectious diseases [Supplemental Material, Table 1 (available online at http://www.ehponline.org/members/2008/11257/suppl.pdf)] and with more deaths at younger ages, it is also likely that the Bangkok population is exposed to a larger number of other risk factors and may be more susceptible to the risks associated with air pollution. Tsai et al. (2000) reported that exposure levels for indoor and outdoor particulates in shopping areas were underestimated by the ambient monitoring stations in Bangkok, and therefore that the excess risk per air pollutant concentration would be higher than if it were a well-calibrated measurement. The higher ratio of PM_2.5_ (PM ≤ 2.5 μm in aerodynamic diameter) to PM_10_ may suggest that the proportion of smaller particles in the PM_10_ composition in Bangkok is more important and might be more strongly related to adverse health effects than in the other cities (Jinsart et al. 2002).

In all the three Chinese cities, the maximum effects always occurred at lag 0–1 days, except for O_3_ in Shanghai, where maximum effects were recorded at longer lags. The lag pattern is consistent with other reports in demonstrating a maximum at lag 1 day for most pollutants (Samoli et al. 2005, 2006). However, for O_3_, the effect estimates are maximal at longer lags, showing that the pattern is also consistent with the literature (Goldberg et al. 2001; Wong et al. 2001). The lag patterns of SO_2_ and O_3_ in Bangkok are consistent with those of the three Chinese cities; however, the Bangkok lag patterns for NO_2_ and PM_10_, with greater effects at longer lags, are different from those of the three Chinese cities. For the traffic-related pollutants NO_2_ and PM_10_, the effects appear to be stronger, and they also seem to last longer in Bangkok than in the three Chinese cities.

In all cities in the PAPA study, the effects of air pollution are stronger for cardiopulmonary causes than for all natural causes. This is consistent with results from most North American and Western European studies (Anderson et al. 2004; Samet et al. 2000) and supports the validity of the estimates from the present study. In addition, the effects of the four single pollutants appear to be stronger at older ages than at younger ages, particularly in Bangkok, which may have a more susceptible population than the three Chinese cities. The stronger effects at older ages for these pollutants support the validity of our estimates.

As expected, the exclusion of high levels of PM_10_ concentrations from the analysis affects the effect estimates. In the present study, consistent with the literature from North America and Western Europe, exclusion of PM_10_ concentrations greater than the 75th or 95th percentile produces larger estimates in all three Chinese cities. These results suggest that the CR curves might be curvilinear, with the slope less steep at higher concentrations. We cannot explain the opposite findings noted in Bangkok; however, they may be related to the exclusion of readings from one monitor located in a region with both high particulate levels and a fairly susceptible population.

The health effects estimates during the warm season are higher than those with all seasons combined in both Bangkok (excess risk 2.16 vs. 1.25%) and Wuhan (0.81 vs. 0.43%), but those in Hong Kong (0.37 vs. 0.53%) and Shanghai (0.24 vs. 0.26%) were similar or lower. These observations support the hypothesis that the populations in Bangkok and Wuhan, which are less affluent than the other two cities, may be more exposed and susceptible because of less use of air conditioning in summer; this may also explain the generally higher air pollution effects observed in Bangkok and Wuhan than in the other two cities (Long et al. 2007). The lower effect in Hong Kong may also be explained by air mass movements and southerly winds prevalent in the summer. In Wuhan the higher effect may be due to extremely high temperatures in summer. There may also be synergistic effects between PM_10_ and extremely high temperatures on mortality. Nevertheless, further study will be important in understanding how results derived from hotter climates could be extrapolated to cooler climates.

Understanding the shapes of the CR curves is important for environmental public health policy decision making and setting of air quality standards. Comparison across geographic regions is also important in demonstrating causality and how effects estimated from one location can be generalized to others. The CR curves for PM_10_ effects on all natural-cause mortality derived from the present study clearly show that the relationship is linear without a threshold in most of the cities studied, although some nonlinear relationships appear in Shanghai. Thus our estimates are consistent with a linear model without threshold, a finding in most North American and Western European studies (Daniels et al. 2000; Pope and Dockery 2006; Samoli et al. 2005). The CR relation of a pollutant would be affected by the method used, the susceptibility of the population being investigated, the toxicity of the pollutant, and the weather and social conditions with which the pollutant may interact.

**Figure 2:**
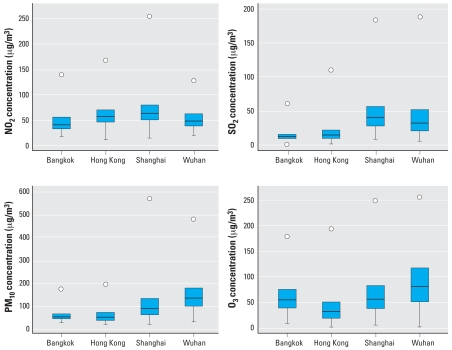
Box plots of the air pollutants for the four cities. Boxes indicate the interquartile range (25th percentile–75th percentile); lines within boxes indicate medians; whiskers and circles below boxes represent minimum values; and circles above boxes indicate maximum values.

**Figure 3:**
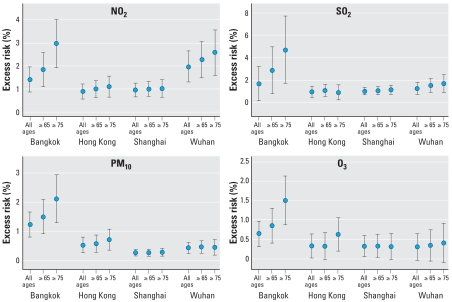
Excess risk (%) of mortality [point estimates (95% CIs)] for a 10-μg/m^3^ increase in average concentration of lag 0–1 days for three age groups.

**Figure 4:**
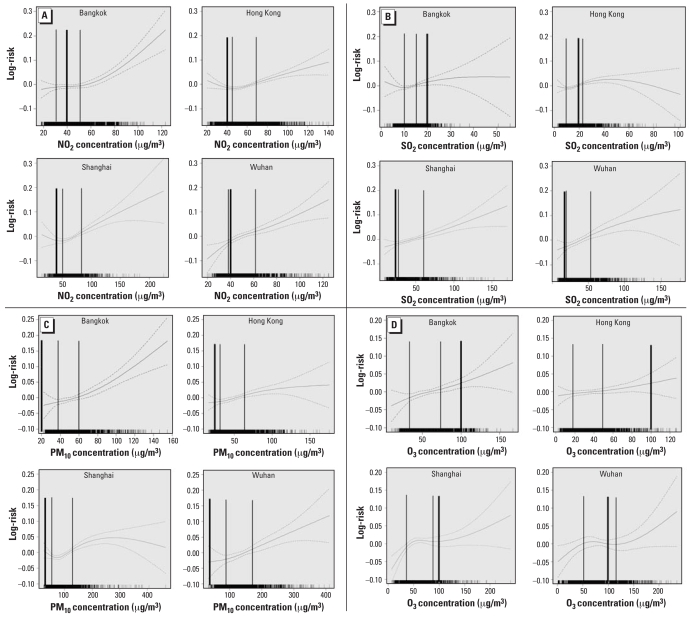
CR curves for all natural-cause mortality at all ages in all four cities for the average concentration of lag 0–1 days for NO_2_ (*A*), SO_2_ (*B*), PM_10_ (*C*), and O_3_ (*D*). The thin vertical lines represent the IQR of pollutant concentrations. The thick lines represent the WHO guidelines (WHO 2005) of 40 μg/m^3^ for 1-year averaging time for NO_2_ (*A*), 20 μg/m^3^ for 24-hr averaging time for SO_2_ (*B*), 20 μg/m^3^ for 1-year averaging time for PM_10_ (*C*), and 100 μg/m^3^ for daily maximum 8-hr mean for O_3_ (*D*).

In the present study, effect estimates for PM_10_ are comparable, whereas those for gaseous pollutants, particularly for NO_2_, are higher than those in the West. One postulation for the higher effect estimates may be related to their correlation with particulate pollutant [correlation between PM_10_ and NO_2_ ranging from 0.71 to 0.85; Supplemental Material, Table 3 (available online at http://www.ehponline.org/members/2008/11257/suppl.pdf)]. However for the three Chinese cities, the estimates for effects of NO_2_ remain robust after adjustment for PM_10_ (Supplemental Material, Figure 2A); whereas those of the PM_10_ effects were attenuated (Supplemental Material, Figure 2B). But for Bangkok, the change in effect estimates for the two pollutants after adjustment for the other as a copollutant are opposite of those for the three Chinese cities. Thus in Asian cities, the observed effects of gaseous pollutants may not necessarily be related to their covariation with a particulate pollutant. Further research is needed to clarify the effects of copollutants.

### Limitations

Among the major limitations of our study was the difference in monitoring locations among the cities. In densely populated cities such as Hong Kong and Shanghai, the monitors tend to be close to major roadways, whereas in Bangkok and Wuhan the monitors are located farther from major pollutant sources. Thus, it is difficult to determine the true effects and to compare our results both within the PAPA cities and with previous studies. In addition, the specific components of particulate responsible for the observed health effects have not been elucidated. Such identification will aid in targeting and prioritizing future pollution control efforts. Also, information about potential effect modifiers (e.g., time spent outdoors, use of air conditioning, residential distance to roadways, housing construction, comorbidity in the population) varied in its availability and quality among the cities, making it difficult to explain quantitative differences among the PAPA cities.

## Conclusion

Effects of particulate pollutants in Asian cities are similar to or greater than those observed in most North American and Western European cities in spite of large differences in concentrations; similarly, effects of gaseous pollutants in Asian cities are as high or higher. The methodology adopted and developed in the PAPA study could be used for other countries preparing to conduct air pollution studies. In addition, results from PAPA studies can be used in Asian and other cities for health impact assessment. Finally, further efforts are needed to understand the socioeconomic and demographic factors that might modify the effects of air pollution.

## Figures and Tables

**Table 1:** Summary statistics of daily mortality counts.

	Mean ± SD				Minimum, maximum			
	Bangkok	Hong Kong	Shanghai	Wuhan	Bangkok	Hong Kong	Shanghai	Wuhan
All natural causes								
All ages	94.8 ± 12.1	84.2 ± 12.8	119.0 ± 22.5	61.0 ± 15.8	29, 147	48, 135	51, 198	25, 213
≥ 65 years	34.3 ± 6.7	65.4 ± 11.6	99.6 ± 20.6	43.8 ± 13.4	13, 63	34, 113	46, 175	18, 159
≥ 75 years	21.3 ± 5.2	43.6 ± 9.5	71.5 ± 16.7	25.7 ± 9.5	6, 50	17, 82	33, 129	6, 106
Cardiovascular	13.4 ± 4.3	23.8 ± 6.5	44.2 ± 11.0	27.8 ± 8.8	1, 28	6, 54	11, 85	8, 94
Respiratory	8.1 ± 3.1	16.2 ± 5.2	14.3 ± 6.4	7.0 ± 5.8	1, 20	3, 34	3, 45	0, 125

Study period: Bangkok, 1999–2003; Hong Kong, 1996–2002; and Shanghai and Wuhan, both 2001–2004.

**Table 2:** Summary statistics of air pollutant concentrations and meteorological conditions.

	Mean				Median				IQR				Minimum, maximum			
	Bangkok	Hong Kong	Shanghai	Wuhan	Bangkok	Hong Kong	Shanghai	Wuhan	Bangkok	Hong Kong	Shanghai	Wuhan	Bangkok	Hong Kong	Shanghai	Wuhan
NO_2_ (μg/m^3^)	44.7	58.7	66.6	51.8	39.7	56.4	62.5	47.2	23.1	24.4	29.0	24.0	15.8, 139.6	10.3, 167.5	13.6, 253.7	19.2, 127.4
SO_2_ (μg/m^3^)	13.2	17.8	44.7	39.2	12.5	14.7	40.0	32.5	5.5	12.6	28.7	30.8	1.5, 61.2	1.4, 109.3	8.4, 183.3	5.3, 187.8
PM_10_ (μg/m^3^)	52.0	51.6	102.0	141.8	46.8	45.5	84.0	130.2	20.9	34.9	72.0	80.2	21.3, 169.2	13.7, 189.0	14.0, 566.8	24.8, 477.8
O_3_ (μg/m^3^)	59.4	36.7	63.4	85.7	54.4	31.5	56.1	81.8	36.2	31.6	45.1	67.4	8.2, 180.6	0.7, 195.0	5.3, 251.3	1.0, 258.5
Temperature (°C)	28.9	23.7	17.7	17.9	29.1	24.7	18.3	18.5	1.8	8.0	14.4	16.3	18.7, 33.6	6.9, 33.8	−2.4, 34.0	−2.5, 35.8
RH (%)	72.8	77.9	72.9	74.0	73.0	79	73.5	74.0	10.8	10.0	15.5	19.0	41.0, 95.0	27, 97.0	33.0, 97.0	35.0, 99.0

Abbreviations: IQR, interquartile range; RH, relative humidity. NO_2_, SO_2_, and PM_10_ are expressed as 24-hr averages, and O_3_ is an 8-hr average.

**Table 3:** Excess risk (ER; %) of mortality (95% CI) for a 10-μg/m^3^ increase in the average concentration of lag 0–1 days by main effect estimates of individual cities and combined random effects.

		Bangkok		Hong Kong		Shanghai		Wuhan		Random effects (4 cities)		Random effects (3 Chinese cities)	
	Pollutant	ER	95% CI	ER	95% CI	ER	95% CI	ER	95% CI	ER	95% CI	ER	95% CI
All natural causes	NO_2_	1.41	0.89 to 1.95	0.90	0.58 to 1.23	0.97	0.66 to 1.27	1.97	1.31 to 2.63	1.23	0.84 to 1.62*	1.19	0.71 to 1.66*
(all ages)	SO_2_	1.61	0.08 to 3.16	0.87	0.38 to 1.36	0.95	0.62 to 1.28	1.19	0.65 to 1.74	1.00	0.75 to 1.24	0.98	0.74 to 1.23
	PM_10_	1.25	0.82 to 1.69	0.53	0.26 to 0.81	0.26	0.14 to 0.37	0.43	0.24 to 0.62	0.55	0.26 to 0.85#	0.37	0.21 to 0.54
	O_3_	0.63	0.30 to 0.95	0.32	0.01 to 0.62	0.31	0.04 to 0.58	0.29	−0.05 to 0.63	0.38	0.23 to 0.53	0.31	0.13 to 0.48
Cardiovascular	NO_2_	1.78	0.47 to 3.10	1.23	0.64 to 1.82	1.01	0.55 to 1.47	2.12	1.18 to 3.06	1.36	0.89 to 1.82	1.32	0.79 to 1.86
	SO_2_	0.77	−2.98 to 4.67	1.19	0.29 to 2.10	0.91	0.42 to 1.41	1.47	0.70 to 2.25	1.09	0.71 to 1.47	1.09	0.72 to 1.47
	PM_10_	1.90	0.80 to 3.01	0.61	0.11 to 1.10	0.27	0.10 to 0.44	0.57	0.31 to 0.84	0.58	0.22 to 0.93**	0.44	0.19 to 0.68
	O_3_	0.82	0.03 to 1.63	0.62	0.06 to 1.19	0.38	−0.03 to 0.80	−0.07	−0.53 to 0.39	0.37	0.01 to 0.73	0.29	−0.09 to 0.68
Respiratory	NO_2_	1.05	−0.60 to 2.72	1.15	0.42 to 1.88	1.22	0.42 to 2.01	3.68	1.77 to 5.63	1.48	0.68 to 2.28	1.63	0.62 to 2.64*
	SO_2_	1.66	−3.09 to 6.64	1.28	0.19 to 2.39	1.37	0.51 to 2.23	2.11	0.60 to 3.65	1.47	0.85 to 2.08	1.46	0.84 to 2.08
	PM_10_	1.01	−0.36 to 2.40	0.83	0.23 to 1.44	0.27	−0.01 to 0.56	0.87	0.34 to 1.41	0.62	0.22 to 1.02	0.60	0.16 to 1.04
	O3	0.89	−0.10 to 1.90	0.22	−0.46 to 0.91	0.29	−0.44 to 1.03	0.12	−0.89 to 1.15	0.34	−0.07 to 0.75	0.23	−0.22 to 0.68

*p*-Values (homogeneity test):

* 0.01 < *p* ≤ 0.05;

** 0.001 < *p* ≤ 0.01; and

# *p* ≤ 0.001.

**Table 4:** Excess risk (ER; %) of mortality (95% CI) for a 10-μg/m^3^ increase in the average concentration of lag 0–1 days by sensitivity analysis for PM_10_ effects with variation in concentration levels, stations, seasons and methods.

	ER				Random effect (4 cities)			Random effect (3 Chinese cities)		
All natural causes, all ages	Bangkok	Hong Kong	Shanghai	Wuhan	ER	95% CI	*p-*Value	ER	95% CI	*p-*Value
Main analysis	1.25	0.53	0.26	0.43	0.55	0.26–0.85	_#_	0.37	0.21–0.54	NS
Omit PM_10_ > 95 percentile	0.82^a^	0.75^a^	0.28	0.52^a^	0.53	0.27–0.78	*	0.47^a^	0.21–0.73	*
Omit PM_10_ > 75 percentile	0.73^a^	0.89^a^	0.36^a^	0.70^a^	0.53	0.29–0.78	NS	0.55^a^	0.24–0.85	NS
Omit PM_10_ > 180 μg/m^3^	1.25	0.54	0.22	0.73^a^	0.65	0.24–1.06	_#_	0.46^a^	0.15–0.76	*
Omit stations with high traffic source	1.18	0.54	0.25	0.45	0.55	0.26–0.85	_#_	0.38	0.20–0.57	NS
Warm season defined by simple dichotomous variables	2.16^a^	0.37^a^	0.24	0.81^a^	0.86^a^	0.11–1.60	_#_	0.43	0.10–0.76	NS
Add temperature at lag 1–2 days	1.06	0.43	0.23	0.48	0.51	0.23–0.79	_#_	0.36	0.18–0.53	NS
Add temperature at lag 3–7 days	0.96^a^	0.36^a^	0.15^a^	0.34^a^	0.35^a^	0.14–0.57	**	0.25^a^	0.10–0.40	NS
Daily PM_10_ defined by centering	1.20	0.53	0.26	0.42	0.54	0.26–0.82	_#_	0.37	0.21–0.53	NS
Natural spline with (8, 4, 4) df	1.23	0.54	0.28	0.38	0.54	0.26–0.81	_#_	0.36	0.23–0.49	NS
Penalized spline	1.20	0.48	0.28	0.39	0.52	0.26–0.77	_#_	0.34	0.23–0.45	NS

NS, not significant.

a ER changed > 20% from the main analysis. *p*-Values (homogeneity test):

* 0.01 < *p* ≤ 0.05;

** 0.001 < *p* ≤ 0.01;

# *p* ≤ 0.001.
